# An assessment of nutrition information on front of pack labels and healthiness of foods in the United Kingdom retail market

**DOI:** 10.1186/s12889-021-10255-4

**Published:** 2021-02-08

**Authors:** D. A. Ogundijo, A. A. Tas, B. A. Onarinde

**Affiliations:** National Centre for Food Manufacturing, University of Lincoln, Holbeach, PE12 7PT UK

**Keywords:** Nutrition labelling, Front of pack, Nutrient profiling, Healthiness of foods

## Abstract

**Background:**

Front of pack nutrition labelling is part of United Kingdom government’s programme of activities aiming to tackle diet-related diseases. There are several front of pack labelling formats available and they differ in the information they deliver. This study assessed the frequency of usage of front of pack food labelling systems on food products in the United Kingdom grocery market. It also measured the healthiness of some foods in the online market by categorising them according to their nutrient contents.

**Methods:**

Five hundred food products in five categories [(1) cereals and cereal products, (2) dairy products, (3) beverages, (4) packaged meats and meat products, and (5) pre-packaged fruits and vegetables] from three main United Kingdom retail websites were investigated. A simple random sampling method was used for product selection according to the categories on the retailers’ websites. The healthiness of foods was also assessed by categorising them into ‘healthier’, ‘moderately healthy’ and ‘least healthy’ based on fat, saturated fat, salt and sugar contents.

**Results:**

The total number of label types assessed comprises 19.6% of Guideline Daily Amounts or Reference Intakes and 43.8% had a combination of Traffic Light and Reference Intakes (hybrid label). Slightly over a quarter (27.4%) of products included nutritional information in a grid or table, 3.4% of the foods had two of any of the following: Health Logo, Reference Intakes and Traffic Light labels, and 5.8% did not have any Reference Intakes, Traffic Light, Health Logo or Hybrid label. Most of the foods assessed were manufactured in the United Kingdom with only 30.8% imported from 32 countries across four continents.

**Conclusions:**

Traffic Light and Guideline Daily Amounts were the most used front of pack labelling formats on the assessed food product. A higher number of assessed products belonged to the “moderately healthy” and “healthier” categories than the “least healthy”. The imported foods that were found in the United Kingdom retail market showed that food choices could be made from the diverse food types.

## Background

Research has shown an increase in the level of diet-related non-communicable diseases (NCDs) such as obesity, overweight, type 2 diabetes and cardiovascular disease. Although these diseases have been common in high-income countries since the end of the twentieth century, recently developing countries are beginning to face similar threats owing to increased consumption of highly processed and energy-dense foods [1–6]. In a document published by the World Health Organization (WHO), total global deaths attributed to NCDs was 78% [7]; this high incidence of NCDs has initiated efforts by the WHO and Food Standards Agency (FSA) to reduce the global health effects of NCDs by 2030 [8, 9].

Nutritional labelling is used as a tool to inform the general public about the healthiness of foods, protect consumers against unsafe foods and prevent manufacturers from making false, deceptive and misleading claims [10, 11]. Nutrition labels can either be on the back of pack (BOP) or front of a pack (FOP). In BOP labels, energy (in kJ or kcal) and the amounts of fat, saturated fat, carbohydrates, sugars, protein and salt (in g) are indicated. BOP was the most prevalent and mandatory label format worldwide in 2014 [11, 12].

The FOP traffic-light (TL) system that was recommended by the UK government for healthier food choices identifies four nutrients in addition to energy values. It categorises foods key nutrient contents as ‘low’, ‘medium’ and ‘high’, represented with green, amber or red, respectively. Moreover, the Guideline Daily Amounts (GDA), which is also referred to as Reference Intake (RI),^1^ presents the recommended number of calories, fat, saturated fat, sugar or salt per day that an average individual should consume, beyond which the consumption may have an adverse effect on health. In the TL system, the United Kingdom’s FSA postulated standards for categorising foods according to nutrient levels per 100 g or 100 ml as shown in Table 1a and b [13, 14]. The criteria^2^ were proposed as a standard in agreement with government agencies in England, Scotland, Wales and Northern Ireland. This was implemented owing to an increase in the reported incidence of diet related NCDs [14]. Grunert et al. [15], Stones [16] and Hodgkins [17] highlighted that the UK’s use of FOP was the highest in Europe.

**Table 1:** Criteria for categorising foods into heathier food products – LOW (green), products with medium nutrients – MEDIUM (amber) and least healthy products – HIGH (red). (A) Per 100 g of food (B) Per 100 ml of drinks (FSA, 2016)

A			
	LOW	MEDIUM	HIGH
Colour code	Green	Amber	> 25% of RIs
Fat	≤ 3.0 g/100 g	> 3.0 g to ≤17.5 g/100 g	> 17.5 g/100 g
Saturated fat	≤ 1.5 g/100 g	> 1.5 g to ≤5.0 g/100 g	> 5.0 g/100 g
(Total) Sugars	≤ 5.0 g/100 g	> 5.0 g to ≤22.5 g /100 g	> 22.5 g/100 g
Salt	≤ 0.3 g/100 g	> 0.3 g to ≤1.5 g/100 g	> 1.5 g/100 g
B			
	LOW	MEDIUM	HIGH
Colour code	Green	Amber	> 12.5% of RIs
Fat	≤ 1.5 g/100 ml	> 1.5 g to ≤8.75 g/100 ml	> 8.75 g/100 ml
Saturated fat	≤0.75 g/100 ml	> 0.75 g to ≤2.5 g/100 ml	> 2.5 g/100 ml
(Total) Sugars	≤ 2.5 g/100 ml	> 2.5 g to ≤11.25 g/100 ml	> 11.25 g/100 ml
Salt	≤ 0.3 g/100 ml	> 0.3 g to ≤0.75 g/100 ml	> 0.75 g/100 ml

This study assesses the frequency of usage of colour coded TL, GDA, a hybrid of colour coded TL and GDA (HYD), and HL food rating systems on food products in the UK grocery market. The study also measures the healthiness of some foods in the online UK market by categorising them into ‘low’, ‘medium’ and ‘high’ according to their nutrient profiles based on the FSA’s recommendation on their salt, sugar and fat thresholds. These categories reflect the level of healthiness of the assessed food products.

## Methods

The nutrient profiles of 500 foods comprising five categories, (1) cereals and cereal products, (2) dairy products, (3) beverages, (4) packaged meats and meat products, and (5) pre-packaged fruits and vegetables were obtained from three of the ‘Big Four’ supermarkets with the highest grocery market share in the UK between August 2012 and August 2019 [18]. These categories were selected according to the classifications based on food industry ingredients [19], retailers` categories [20] and household usage [21]. Non-packaged or ‘loose’ meat, fruits and vegetables were not included because preference was given to products with either FOP and or BOP label. The selection of the products was not based on brand or manufacturer’s name, price, name of product nor perceived healthiness of the product by the researcher.

The retail market was chosen because of the recommendations of Rayner et al. [22] that not many studies have been done by exploring food retailers’ websites. The authors emphasised that it is important for researchers to monitor the components of different labelling formats in the retail market and their likelihood in affecting health-related food choices. Similar to Rayner et al. [22] recommendation, Stones [16] also reiterated the significance of studying the use of nutrition labels in an online food environment, due to the increase in online shopping and the prevalence of diet-related illnesses across the globe.

Assessments were carried out on fat, saturated fat, sugar and salt, which are the four most declared nutrients on FOP nutrition labels. The following information for each product was included in an excel spreadsheet: country of origin, serving/preparation instructions, declaration of allergens, type of labelling tool used, BOP nutrient amounts, health and nutrition claims, product weight, expiry dates (use by date and/or best before date), storage information, description of the product or the statement of identity and dietary information provided by the manufacturer. The data obtained were sorted by the excel spreadsheet ‘sort and filter’ tool and organised for easy categorisation when necessary.

### Product selection and assessment

A simple random sampling method was used to select products that fall into the categories of cereals and cereal products, dairy products, beverages, packaged meats and meat products, and pre-packaged fruits and vegetables from the three websites.^3^ The products in the categories were chosen as stated on the retailers’ (Sainsbury’s [23], Tesco [24] and Morrisons [25]) websites, and which are regularly consumed in most households. All the products that were randomly picked were from the same category listed on the websites. Every sample chosen was a representative of the products in each category. Although this type of probability sampling method may be time consuming, it is one of the most reliable methods that eliminate selection bias [26]. Any product that belong to the categories has a likelihood of being chosen and examples of the chosen products are as follows:

(1) Breakfast cereals. This category included common food products that are referred to as ‘breakfast cereals’ in the UK. Examples are porridges, chocolate shreddies, rice crispies, cookie crisp, sugar puffs, corn flakes, bran flakes, granola, bars and frosties.

(2) Dairy products. This category included products with a minimum of 40% milk. Examples are milk, yoghurt, cream, cheese, kefir, whey, fromage-frais.

(3) Beverages. The beverages sampled were (a) non-alcoholic drinks Group A, e.g. coffee, tea, chocolate, cocoa products, (b) non-alcoholic drinks Group B, e.g. carbonated drinks, fizzy drinks (c) alcoholic drinks e.g. beer, cider, spirits, vodka, gin, tequila, rum, whisky, brandy, etc.

(4) Meats (pre-packaged meats and meat products). These were not specific to any type.

(5) Fruits and vegetables (pre-packed fruits and vegetables). These were either fresh, frozen or dried.

The food products included must have either the retailer or the manufacturer label when randomly selected. Attention was given to the type of FOP (TL, GDA, HL, or a combination of these) or BOP label format used on the product, the presence of nutrient or health claims, country of origin, list of ingredients, declaration of allergens or dietary information, presence of product weight and the declaration of the amount of calories in kilocalories, fat, saturated fat, sugar, and salt per 100 g or per 100 ml of the food products. The assessment focused on the types of nutrition labelling tools used, and the categorisation of the foods is based on the nutrient profiles of key nutrients. Imported foods and food products without nutrition labelling or dietary information were excluded.

Since foods were randomly selected, the food products that were assessed were not limited by origin. The products originated from 33 different countries in Europe, America, Asia and Africa, namely the UK, France, Italy, the Netherlands, Spain, Austria, Ireland, Belgium, Cyprus, Denmark, Germany, Romania, Greece, Luxembourg, Poland, Sweden, Argentina, Costa Rica, USA, Chile, Australia, Brazil, Egypt, India, Indonesia, Kenya, Mexico, Philippines, New Zealand, Poland, Tunisia, Israel and Turkey.

### Criteria for categorisation

In this study, foods are categorised as ‘high’, ‘medium’ or ‘low’ according to the criteria of FSA [14] by assessing their fat, saturated fat, sugar and salt content (Table 1). High, medium and low contents are matched with colours; red, amber and green, respectively. Red means the food is high in one of these and should be eaten less often or in smaller amounts. Amber means the food contains medium amount (neither high nor low) and may be eaten more frequently. Green indicates that the food is low in these nutrients and therefore is a healthier choice. This categorisation of food products according to their nutrient contents with implications on public health is referred to as ‘Nutrient profiling’ [27]. Although a food product may contain a good amount of nutrients in varying contents, it has been established by Drewnowski et al. [28] that attention must also be given to the number of calories, which has a direct effect on the health of the consumers.

## Results

Of the 500 products that were assessed, 346 (69.2%) were produced in the UK. As shown in Table 2, the country of origin of 14 (2.8%) food products were not specified, nine (1.8%) were said to be made in the EU and no specific country was declared on five (1.0%) products. The country with the highest number of imported foods from outside Europe is the United States, with 21 (4.2%) food products. Products from the UK were represented by 26.0% meat and meat products, 19.6% of the beverages, 19.4% of the dairy products, 18.5% of the breakfast cereals and 16.5% of the fruits and vegetables.

**Table 2:** Assessed samples with nutrition labels grouped according to country of origin (*n =* 500)

S/N	Country	HYD	GDA	GDA & Health logo	HYD & Health logo	Nutrition Information grid	Absent	Total sample number	%
1	United Kingdom	169	77	3	5	82	10	346	69.2
2	United States	1	1			17	2	21	4.2
3	Republic of Ireland	6	2			4		12	2.4
4	Italy	7	1		1	2		11	2.2
5	France	6	1			3	2	10	2
6	Spain	4	3			2	1	9	1.8
7	EU*	1	4			4		9	1.8
8	Netherlands	6	1			1	5	8	1.6
9	New Zealand	4	2				2	6	1.4
10	Germany	1	1			3		5	1
11	Poland		1		2	2		5	1
12	Denmark	1				3		4	0.8
13	Greece	1	1			1		3	0.6
14	Turkey				3			3	0.6
15	Belgium	1	1					2	0.4
16	Argentina		2					2	0.4
17	Brazil		2					2	0.4
18	Chile	1				1	1	2	0.4
19	Kenya		1		1			2	0.4
20	Austria					2		2	0.4
21	Sweden		1			1	1	2	0.4
22	Romania	1						1	0.2
23	Luxembourg					1		1	0.2
24	Costa Rica					1		1	0.2
25	Egypt	1						1	0.2
26	India	1						1	0.2
27	Israel					1		1	0.2
28	Philippines	1						1	0.2
29	South Africa					1		1	0.2
30	Tunisia	1						1	0.2
31	Indonesia					1		1	0.2
32	Australia					1		1	0.2
33	Cyprus	1						1	0.2
34	Mexico						1	0	0
35	NP*^a^	3			2			5	1

^a^(*) The product had no specific country of origin.

It was found that 97.1% of the foods that were made in the UK had either one of colour coded TL label, GDA or RIs, Health Logo (HL), declared nutrition information (BOP) or a combination of these, and 48.8% of the assessed UK foods had hybrid (HYD) labels. Only 2.3% of the total foods manufactured in the UK had health logos. Most of the imported foods from the US (3.8%), Italy (2.2%), France (2%), Spain (1.8%), Netherlands (1.6%) and New Zealand (1.4%) had either colour coded TL, GDA or a combination of both. The TL colour coded labelling format was specific to UK manufactured foods, for example, only one of the twenty-one foods from the US had a colour coded TL label.

All breakfast cereals and dairy products stated fat, saturated fat, sugar and salt, 1.0% of the meat products did not state the fat content, and 2.0% of the meat products did not have saturated fat, sugar and salt content declared (Table 3). The declarations of the key four nutrients are presented in Tables 3 and 4. Fat, saturated fat, sugar and salt were not stated on 36.0% of the labels of the beverages (hot drinks, fizzy drinks, wines, gins and confectionery such as cocoa drinks). In addition, 33.2% of the foods did not have TL, GDAs nor nutrition table or grid, which were either absent or not visible to online shoppers.

**Table 3:** Frequency of nutrients declared per food categories (*n =* 500)

Nutrient	Cereals	Dairy	Meats	Beverages	Fruits and vegetables	Total	Mean
Fat	100	100	99	64	100	463	92.6
Saturated fat	100	100	98	64	98	460	92.0
Sugar	100	100	98	64	100	462	92.4
Salt	100	100	98	64	97	459	91.8

**Table 4:** Frequency of nutrients not declared per food categories (*n =* 500)

Nutrient	Cereals	Dairy	Meats	Beverages	Fruits and vegetables	Total	Mean
Fat	0	0	1	36	0	37	7.4
Saturated fat	0	0	2	36	2	40	8.0
Sugar	0	0	2	36	0	38	7.6
Salt	0	0	2	36	3	41	8.2

Moreover, 36.0% of the beverages did not have these four nutrients, this may be as a result of voluntary declaration of these nutrients on beverages according to Articles 16(4) and 44(1)(b) Annex (V) of the (EU) 1169/2011 regulation [29] on food labelling. In this regulation, exemptions for declaration of these nutrients are given to beverages such as whole or milled coffee beans, single ingredient products, fruit infusions, tea, fermented vinegars and beverages with more than 1.2% v/v alcohol.

The categorisation of food products based on the amount of their nutrients are presented in Table 5. As it can be observed in Table 5, out of the total foods assessed, 15.6% were high in fat, 21.4% were ‘high’ in saturated fat, 14.2% were high in sugar and 5.8% were high in salt, which renders them least healthy. Only 16% of the meat and meat products randomly sampled were high in fat and 38% were high in saturated fat.

**Table 5:** Percentages of food products classed as ‘High’ – Least healthy, based on the FSA (2016)

Food category	Fat		Saturated fat		Sugar		Salt	
	n	(%)	n	(%)	n	(%)	n	(%)
Cereals	16	20.5	18	16.8	35	49.3	5	17.2
Dairy	36	46.2	40	37.4	10	14.1	12	41.4
Beverages	3	3.8	6	5.6	11	15.5	0	0.0
Meats	16	20.5	38	35.5	0	0.0	12	41.4
Fruits and vegetables	7	9.0	5	4.7	15	21.1	0	0.0
Total	78	100.0	107	100.0	71	100.0	29	100.0
Overall %	500	15.6	500	21.4	500	14.2	500	5.8

Some of the dairy products sampled had the greatest proportion of products with high fat and saturated fat. The dairy products assessed include milk of various types such as whole milk, skimmed milk and buttermilk, yoghurt, cheese and ice-cream. These had the highest amounts of nutrients, 36.0% high in fat, 40.0% high in saturated fat, 10.0% high in sugar and 12.0% high in salt. Of the assessed breakfast cereals, 16.0% was high in fat, 18.0% high in saturated fat, 35.0% high in sugar and 5.0% high in salt.

Also, in the breakfast cereals category, 61.0% of the products had the amount of fat that fall into the medium category (Table 6), 24.0% had medium saturated fat, 48.0% had medium sugar and 46.0% had medium salt content. The meats assessed had the highest number of products with medium nutrients. Among these, 73.0% of meats had medium fat, 45.0% had medium saturated fat, 5.0% medium sugar and 44.0% of the products had medium salt. In contrast, beverages had the lowest number of products with ‘medium’ nutrients. Only 3.0% had medium fat, 1.0% medium saturated fat, 32.0% medium sugar and 6.0% medium salt. When all the products were considered, 37.2% had a medium amount of fat, 22.0% medium saturated fat, 33.8% medium sugar and 25.2% medium salt content.

**Table 6:** Percentages of food products with declared nutrients classed as ‘Medium’ – Moderately healthy, based on the FSA (2016)

Food category	Fat		Saturated fat		Sugar		Salt	
	n	(%)	n	(%)	n	(%)	n	(%)
Cereals	61	34.3	24	21.8	48	28.4	46	36.5
Dairy	33	18.5	33	30.0	46	27.2	19	15.1
Beverages	3	1.7	1	0.9	32	18.9	6	4.8
Meats	73	41.0	45	40.9	5	3.0	44	34.9
Fruits and vegetables	8	4.5	7	6.4	38	22.5	11	8.7
Total	178	100.0	110	100.0	169	100.0	126	100.0
Overall %	500	35.6	500	22.0	500	33.8	500	25.2

Among the 500 foods assessed, 41.4% had low fat, 48.6% had low saturated fats, 44.4% had low sugar and 52.0% had low salt levels (Table 7). The breakfast cereals have the least number of the overall products with low amount of sugar (7.7%), 9.5% were beverages, 19.8% were dairy products and 41.8% were meats. Higher number of dairy products (15.0%) were found to contain low fat when the numbers with low nutrients was compared. Only 11.1% of the products in the cereal category had low fat, however 23.9% of the cereal products had low saturated fat as compared to 11.1% of the dairy products. Higher number of products in the cereal category (18.6%) were found to have low salt content than the 16.2% in meats and 9.6% of the dairy products.

**Table 7:** Percentages of food products with declared nutrients classed as ‘Low’ – Most healthy, based on the FSA (2016)

Food category	Fat		Saturated fat		Sugar		Salt	
	n	(%)	n	(%)	n	(%)	n	(%)
Cereals	23	11.1	58	23.9	17	7.7	49	18.6
Dairy	31	15.0	27	11.1	44	19.8	25	9.6
Beverages	58	28.0	57	23.5	21	9.5	58	22.4
Meats	10	4.8	15	6.2	93	41.8	42	16.2
Fruits and vegetables	85	41.1	86	35.3	47	21.2	86	33.2
Total	207	100	243	100	222	100	260	100
Overall %	500	41.4	500	48.6	500	44.4	500	52.0

## Discussion

### Nutrition Labelling

**Fig. 1:**
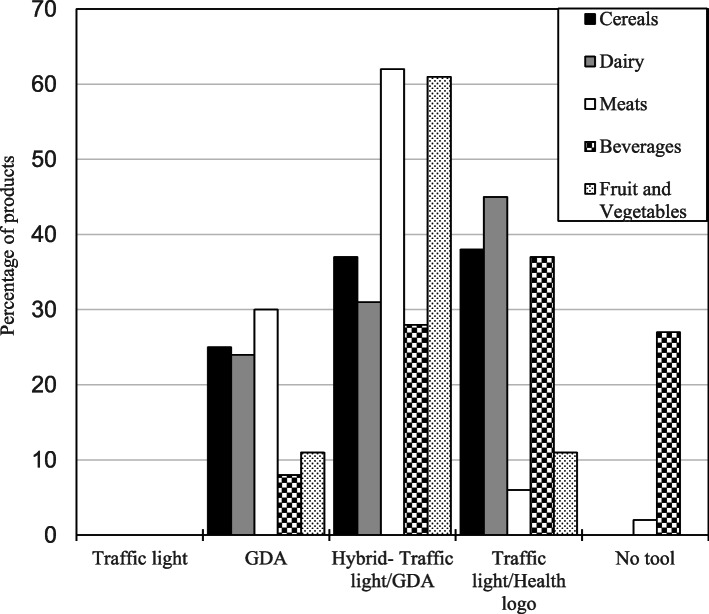
Percentage of occurrence of FOP assessment tools on food categories (*n* = 500). GDA: Guideline Daily Amounts

In this study, HYD label is dominant in the meats and fruits and vegetables categories and was found on 43.8% of all the assessed products. It was seen that the colour coded TL was not used independently in all the products but was used together with GDAs and/or Health Logos. Out of the 346 products that were made in the UK, 72.3% had either GDA, HL or HYD labels (Fig. 1), this is an increase on the 63.0% recorded by Grunert et al. [30]. A greater number of labels with the declaration of nutrition labelling components and more frequent use of FOP nutrition labelling on overall foods were seen in this study when compared to Grunert et al. [15] and Hodgkins [17]. On the contrary, 27.4% of the sampled products was seen to not have TL or RIs displayed on their labels. The absence of the nutrition information on the food products, which is one of the main limitations of the use of nutrition labelling in making food choices, may limit the choice of healthier foods at the point of purchase [16, 31, 32].

All cereals and dairy products had either one or a combination of TL, GDA, HL or hybrid of these types on FOP labels (Fig. 1). It was seen that 27.0% of beverages and 2.0% of meats had neither TL, GDA nor nutrition information. Health logos were predominantly used for fruits and vegetables, and 17.0% of the fruits and vegetables assessed had a hybrid of TL, GDA and health logos. The conceptual and substantive understanding by the consumers of these products was not investigated in this study, but it could be proposed that, based on the types of the labels used, consumers may not have difficulty in choosing healthy food products, should they want to apply labelling information in their purchasing decision. This is because 66.8% of the products have either symbol or colour in the form of TL label format, GDA labels or health logos, which Kelly and Jewell [33] pinpointed as catalysts for making healthier food choices.

It has been indicated that consumers will be likely to have a good understanding of nutrition labelling with colour, exegetical words or symbols [33, 34]. FOP labels have been reported to augment the BOP label information and provide consumers with interpretive symbols, colours or logos to assess a product’s overall healthiness [35, 36]. The use of high/medium/low to represent the nutrient amounts on the nutrition labels (instead of using percentages) has improved the understanding of the consumers [37]. The proportion of the products with FOP labelling in this study was consistent with our expectation that FOP TL labels should be dominant on foods in the UK market as compared with the other kinds that are used elsewhere, such as the Australasia’s Health Star Rating (HRS) system [38] and the Chilean warning label (WL) model [39].

This study indicates that colour coded TL labels and GDA are the most common labelling formats used. The inclusion of nutritional labels may influence larger number of people across many countries, only when consumers decide to use this information to make healthy food choices. Therefore, creating awareness for the importance of understanding and implementing the information on nutritional labels is equally important. Researchers are keen to create such awareness via educational interventions [40, 41].

The non-declaration of specific country of origin on 2.8% (lists with asterisks) of the assessed foods in Table 2 may affect the reliability of nutrition information presented on the labels [33]. This would be one of the major changes to how foods and drinks would be labelled in the UK from 1 October 2022, when it would become a law that foods and drinks labelled as ‘origin EU’ would not be found in the grocery market in the UK.

### Least healthy products

The consumption of foods in these least healthy categories over a prolonged period may have negative effects on the health of an individual. For example, Harvard Medical School [42] and Payne, Piernas and Aveyard [43] emphasised that the consumption of salt (sodium) above the recommended amounts may lead to a rise in blood pressure and heart attack. Although 3.0% of beverages in this study were high in fat, 6.0% high in saturated fat and 11.0% high in sugar, none was high in salt, and this was the same for the fruits and vegetables with zero ‘high level’ salt. These results are consistent with our expectations.

It is unusual to eat a diet free of saturated fat, but a reduction in the consumption of trans fats and saturated fats has been highly recommended through epidemiological data and studies such as Kris-Etherton, Petersen and Van [44], German and Dillard [45] and Hu and Willet [46]. Consuming a diet low in saturated and trans-fats is understood to be one of the ways of preventing cardiovascular diseases, which are reported to be the source of one death occurring in every four minutes in England [47].

It can be seen from our results that despite efforts made by Public Health England (PHE) to ensure a reduction in the consumption of added sugar in processed foods, less attention has been given to the amount of sugars in food products other than beverages, a finding supported by Amoutzopoulos et al. [48]. Bandy, Scarborough and Harrington [49] also highlighted that efforts to reduce added sugar by the soft drinks manufacturing industries should be channelled towards other types of food products such as snacks and breakfast cereals.

### Products with medium nutrient content

When a product contains a ‘medium or moderate’ amount of a nutrient, this implies that its consumption is less likely to affect consumer health negatively. The ability of consumers to be aware of the effect of a food product on their health depends on their knowledge of health inferences [50], nutrition knowledge [30] and food quality [51, 52]. In this study, the number of food products with medium nutrient contents are higher than the number with ‘high in nutrients’, but lower than those with ‘low in nutrients’. There may be health implications when food products with medium nutrient contents are consumed on a regular basis; therefore, it is important that consumers eat an overall diet based on the RIs.

### Healthier products

The food products in this category have low amounts of fat, saturated fats, sugar and salt. As consumers become increasingly conscious about their diets, foods low in these ingredients should continue to gain popularity. Valsta, Tapanainen and Männistö [53] recommended the consumption of meat with low saturated fat as a healthier choice, and Askew [54] identified that consumers now prefer to choose any healthier foods to convenience foods. In the assessed food categories, fruits and vegetables had the highest level of nutrients beneficial to consumers. These results align with the global understanding of the healthiness of fruits and vegetables, which have numerous benefits as presented by Slavin and Lloyd [55]. The 49.0% of the breakfast cereals that had low salt content in this study is consistent with the report by Pombo-Rodrigues et al. [56] which measured the quantity of salt and sugar in breakfast cereals in the UK.

### Limitations of this research

Although some online shoppers left reviews about the products assessed, the lack of evidence of use of nutritional labelling when placing orders online is a limitation of this study. This is the same with the limitations (“buts”) of online shopping reported by Yang, Zhao and Wan, [57] and Bansal [58]. In addition, the number of products assessed in this study is relatively small. Therefore, a study using a greater number of products and websites would be helpful in order to cover more product categories. Selection bias was avoided when the assessed products were randomly selected on the websites but there was little control on how the products were listed when the web pages were refreshed.

## Conclusions

TL and GDA were the most used FOP labelling formats on the assessed food products in the UK retail market. The use of colour in TL and table or grid in GDA are easy to understand and could provide consumers with at-a-glance information of the products which they intend to buy during shopping. In the times of outbreak of diseases where physical contact is restricted, such as the recent Covid-19 pandemic when people are encouraged to shop online; or even when shopping in store, these FOP formats could help consumers to reduce the time they spend to read the labels before making decisions on the foods they buy. Incorporating these formats on all food products may be a good initiative to improve public health nutrition. The risks of many diet-related illnesses may be reduced if consumers use of the FOP labelling to make informed decisions to purchase healthier foods. Adopting the use of FOP nutrition labelling formats such as the UK government’s TL and HL systems, may particularly be helpful to tackle the reported increase of diet-related illnesses in low- and medium-income countries.

Our study found out that a higher number of assessed products belonged to the “moderately healthy” and “healthier” categories than the “least healthy”. This could suggest that food manufacturers are now responding to the trends that are driven by the public’s demand for the wider availability of healthier foods.

The imported foods that were found in the UK retail market also showed that food choices could be made from the diverse food types in the grocery markets. If more widespread use of a front of pack label that is going to be valid across the countries is planned for the future, more attention should be given to how information is disseminated on the label, and the use of easy-to-understand unilateral labelling format that uses colour should be encouraged.

## Data Availability

The datasets used and/or analysed during the current study are available from the corresponding author on reasonable request.

## Abbreviations

FSA: Food Standard Agency
EUFIC: European Information Council
NCDs: Non communicable diseases
GDA: Guideline daily amount
TL: Traffic light
HL: Health logo
HYD: Hybrid label (GDA and TL)
FOP: Front of pack
BOP: Back of Pack
RI: Reference Intake
UK: United Kingdom

## Footnotes

1 Reference intake is the maximum amount of nutrient or energy value (8400 kJ/2000 kcal for an average adult) that must be consumed in a day and it is usually expressed in 100 g or 100 ml or per portion (FSA, 2016).

2 https://www.food.gov.uk/sites/default/files/media/document/fop-guidance_0.pdf

3 https://www.sainsburys.co.uk/shop/gb/groceries https://www.tesco.com/groceries/ https://groceries.morrisons.com/browse
